# [D-Leu^1^]MC-LR Has Lower PP1 Inhibitory Capability and Greater Toxic Potency than MC-LR in Animal and Plant Tissues

**DOI:** 10.3390/toxins12100632

**Published:** 2020-10-01

**Authors:** Daniela Sedan, Luciano Malaissi, Cristian Adrián Vaccarini, Ezequiel Ventosi, Martín Laguens, Lorena Rosso, Leda Giannuzzi, Darío Andrinolo

**Affiliations:** 1 Center for Environmental Research (CIM), National Council for Scientific and Technical Research (CONICET), National University of La Plata (UNLP), La Plata 1900, Argentina; lucianomalaissi09@hotmail.com (L.M.); cristianvaccarini670@gmail.com (C.A.V.); dandrinolo@yahoo.com (D.A.); 2 Area of Toxicology, School of Exact Sciences, National University of La Plata (UNLP), La Plata 1900, Argentina; eventosi@hotmail.com (E.V.); betylorena@yahoo.com.ar (L.R.); 3 Pathology B Cathedra, School of Medical Sciences, National University of La Plata (UNLP), La Plata 1900, Argentina; martinlaguens@gmail.com; 4 Research Center in Food Cryotechnology (CIDCA), National Council for Scientific and Technical Research (CONICET), La Plata 1900, Argentina; leda@biol.unlp.edu.ar

**Keywords:** [D-Leu^1^]Microcystin-LR, Microcystin-LR, toxic potency, phosphatase inhibition, *Phaseolus vulgaris*, mice

## Abstract

Two microcystins, MC-LR and [D-Leu^1^]MC-LR, present in La Plata Basin blooms, are differentiated by substitution of D-Alanine for D-Leucine at position 1. Our objective was to evaluate acute toxicity of [D-Leu^1^]MC-LR and MC-LR in mice (N:NIH Swiss) and beans (*Phaseolus vulgaris*). We observed variations in [D-Leu^1^]MC-LR lethal doses with respect to those reported for MC-LR (100 μg/kg), with an increased liver/body weight ratio and intrahepatic hemorrhages in mice exposed to 50–200 μg [D-Leu^1^]MC-LR/kg and slight steatosis after a single 25 μg [D-Leu^1^]MC-LR/kg i.p. dose. Our study in the plant model showed alterations in germination, development, morphology and TBARs levels after a single contact with the toxins during imbibition (3.5 and 15 µg/mL), those treated with [D-Leu^1^]MC-LR being more affected than those treated with the same concentration of MC-LR. Protein phosphatase 1 (PP1) IC_50_ values were 40.6 nM and 5.3 nM for [D-Leu^1^]MC-LR and MC-LR, respectively. However, the total phosphatase activity test in root homogenate showed 60% inhibition for [D-Leu^1^]MC-LR and 12% for MC-LR. In mouse liver homogenate, 50% inhibition was observed for [D-Leu^1^]MC-LR and 40% for MC-LR. Our findings indicate the need for further research into [D-Leu^1^]MC-LR toxicity since together with oxidative stress, the possible inhibition of other phosphatases could explain the differences detected in the potency of the two toxins.

## 1. Introduction

Cyanobacteria are photosynthetic prokaryote organisms that often develop blooms in lakes, ponds, rivers and reservoirs, favored by climate change [1] and freshwater body eutrophication [2,3]. Several cyanobacteria genera have the ability to produce toxins, called cyanotoxins, which vary in their chemical nature, mechanisms of action and target organ. Cyanobacteria can produce several toxins simultaneously, *Microcystis*, *Anabaena*, *Planktothrix*, *Nostoc* and *Anabaenopsis* being some of the cyanobacteria genera frequently linked to microcystins (MCs) production. There are also several strains able to produce different MCs at the same time [4]. MCs, especially MC-LR, are the most frequently found cyanotoxin in blooms around the world. Another MC frequently found in La Plata Basin and Uruguay River (Concordia, Salto Grande Reservoir) among others, is [D-Leu^1^]MC-LR [5,6,7,8,9,10]. These two MCs are cyclic heptapeptides, differing only in the substitution of D-Alanine for D-Leucine at position 1 of the molecule, this moiety being involved in the binding of the toxin to the active site of protein phosphatases. Though still not fully understood, the main action mechanisms of MCs are the inhibition of protein phosphatases, mainly PP1 and PP2A, by covalent binding of the toxin to cysteine residues (Cys), present in the active site of enzyme catalytic subunits [11], and generation of oxidative stress by inducing the production of reactive oxygen species (ROS) [12].

Numerous studies on MC-LR toxicity have been carried out on animals, mainly in rats, mice and fish, where damage has been described not only in liver but also in the kidney, intestinal tract, lung, brain, thymus, heart and immune system of treated animals [13,14,15,16,17,18,19,20,21,22,23,24,25,26,27,28,29]. The damage pattern caused by MC-LR intoxication depends on the dose, time of exposure and route of administration. Based on MC-LR toxicity literature, the damage derived from acute exposure is very different from that occurring in chronic intoxication. Acute exposure damage is characterized mainly by loss of liver architecture, hepatocyte degeneration and intrahepatic hemorrhage followed by hemodynamic shock and death [13,14,15,18]. At the biochemical level, a decrease was observed in antioxidant enzyme activities and an increase in lipid peroxidation, not only in liver but also in kidney [30]. On the other hand, chronic intoxication is characterized by hepatocyte cytosolic vacuolation, single-cell necrosis, fibrosis, apoptosis, tumor promotion and decrease in intestine intraepithelial lymphocytes [16,17,19,25,27,29]. In these cases, there are several biochemical parameters altered in liver, kidney and plasma, such as lipid peroxidation, enzymatic and non-enzymatic components of the antioxidant system (superoxide dismutase, glutathione, α-tocopherol), methemoglobin and lipid profile [25,26,28].

Some plant models have also been used to study the toxic effects of MCs, especially MC-LR. These works usually address exposure by irrigation or spraying plant leaves with contaminated water or cyanobacteria blooms. The damage to the exposed plants is mainly characterized by leaf necrosis and alterations in photosynthesis and plant development linked to the oxidative stress generated [31,32,33,34,35].

Since MC-LR is one of the most toxic and frequently found MCs in freshwater bodies, it has been taken as a model to study the toxic effects of these cyanotoxins. Papers addressing the toxicity generated by other MCs are scarce. [DLeu^1^]MC-LR has been reported in cyanobacteria water bloom collected in Pakowki Lake, Alberta, Canada [7] and in the Patos Lagoon estuary, Brazil [5] and a first overview of its toxicity undertaken. Although information on the toxic effects of [D-Leu^1^]MC-LR is scarce, it has been suggested that [D-Leu^1^]MC-LR could play an eco-physiological role as an antioxidant protector in cyanobacteria [36], or that it could be a possible candidate for the development of an antimycobacterial agent in the treatment of tuberculosis [37].

Considering the action mechanisms attributed to MC, its relationship with the observed damage and the limited information we have on the toxic effects of [D-Leu^1^] MC-LR, further studies are called for to detect differences in the toxic effects produced by the two MC congeners, MC-LR and [D-Leu^1^]MC-LR, commonly found in the La Plata basin. The objective of this work was therefore to evaluate the toxic effects of acute exposure to MC-LR and [D-Leu^1^]MC-LR on an animal (N: NIH Swiss mice) and plant (*Phaseolus vulgaris*) model. We further aimed at assessing related aspects of these toxins’ action mechanisms, such as their inhibitory effects on phosphatases.

## 2. Results

### 2.1. MC-LR and [DLeu^1^]MC-LR Acute Exposure in Mice

Acute exposure studies were performed by intraperitoneal administration of 25, 50, 100 and 200 µg [D-Leu^1^]MC-LR/kg and 100 µg MC-LR/kg doses in N:NIH Swiss mice. In adherence with the guiding principles underpinning the humane use of animals in scientific research, known as the three Rs (Replace, Reduce, Refine) and taking into account that the damage caused by both acute and chronic exposure to a wide range of doses of MC-LR has been extensively studied in various animal models [13,14,15,16,17,18,19,20,21,22,23,24,25,26,27,28,29], we used a single lethal dose of MC-LR in order to have a point of comparison between the acute toxic effects of the two MCs studied. Animals treated with a single administration of 50, 100 and 200 µg [D-Leu^1^]MC-LR/kg and with 100 µg MC-LR/kg died between 212 ± 43 min and 94 ± 11 min post-injection, while animals treated with 25 µg [D-Leu^1^]MC-LR/kg did not die and were sacrificed at 24 h post-injection for histological study. After necropsy, the livers of animals treated with 50, 100 and 200 µg [D-Leu^1^]MC-LR/kg and with 100 µg MC-LR/kg appeared enlarged with a blackish coloration, indicative at the macroscopic level of the presence of intrahepatic hemorrhage. Likewise, the liver/body weight ratio was over 7% in all cases. In the case of animals treated with 25 µg [D-Leu^1^]MC-LR/kg, a liver/body weight ratio of 6.4% was observed, the liver being enlarged but without hemorrhagic characteristics at macroscopic level.

**Figure 1:**
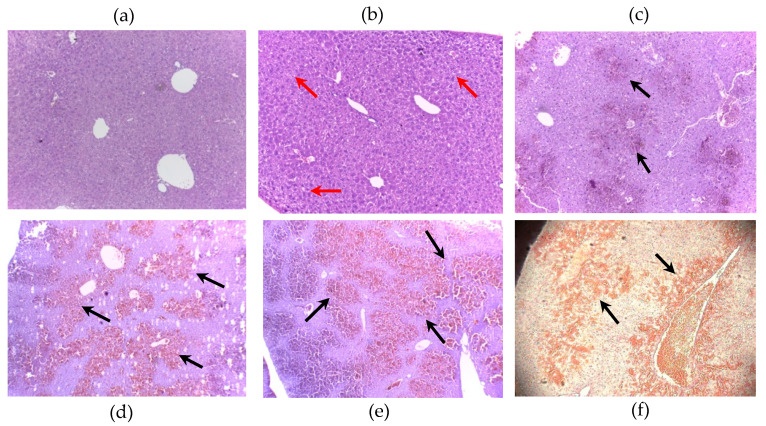
Representative slices Hematoxylin & Eosin-stained liver (100×) from control (**a**) and mice treated with a single i.p. dose of 25 (**b**), 50 (**c**), 100 (**d**), 200 (**e**) µg [D-Leu^1^]MC-LR/kg and 100 µgMC-LR/kg (**f**). Black arrows indicate hemorrhagic areas and red arrows show lipid accumulation areas (slight steatosis).

Hematoxylin & Eosin-stained liver sections showed that all [D-Leu^1^]MC-LR doses (Figure 1b–e) used in this study showed histologic alterations with respect to the control (Figure 1a).

The liver of mice treated with a single i.p. injection of 50 (Figure 1c), 100 (Figure 1d), 200 µg [D-Leu^1^]MC-LR/Kg (Figure 1e) or 100 µg MC-LR/Kg (Figure 1e) showed damage characterized by centrilobular hemorrhages and necrosis with liver cell loss, as opposed to the radiated disposition of hepatocytes typical of control liver (Figure 1a). The histological alterations of the liver generated by the administration of an i.p. dose of 25 µg [D-Leu^1^]MC-LR/Kg (Figure 1b) were different from those produced by the highest doses used and characterized by an increase in lipid accumulation in the centrilobular area, observed as intracytoplasmic microvacuolization corresponding to the development of slight steatosis. No fibrosis, ectasia (vascular congestion), or hemorrhages were observed.

**Figure 2:**
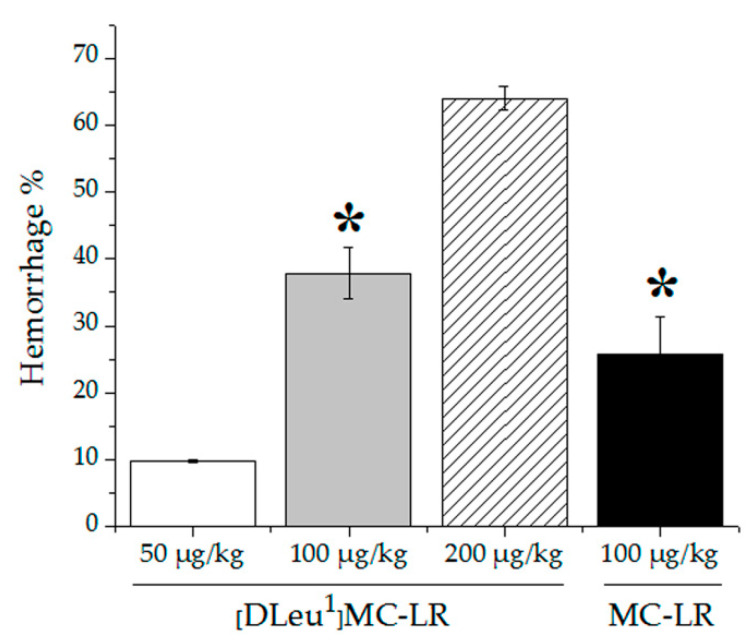
Liver damage in terms of hemorrhage % produced by single i.p. dose of 50 (white bar), 100 (gray bar), 200 (dashed bar) µg [D-Leu^1^]MC-LR/kg and 100 (black bar) µg MC-LR/kg. Note that the hemorrhagic liver area becomes larger as the dose increases and that at the same dose (100 µg/kg) the lesion in the treatment with [D-Leu^1^]MC-LR (37.9 ± 3.8%) is greater than with MC-LR (25.8 ± 5.6%). The results are expressed as Mean ± SD (*N* = 4). (*) indicates significant differences between MC congeners at the same concentration, according to ANOVA test of two independent populations (*p* < 0.05).

Although the damage generated by doses between 50 and 200 µg [D-Leu^1^]MC-LR/Kg and 100 µg MC-LR/Kg had similar characteristics, mainly intrahepatic hemorrhage, it is noteworthy that this damage was dose- and congener-dependent (Figure 2).

In the case of treatments with a single i.p. administration of [D-Leu^1^]MC-LR, the observed damages were thus consistent with 9.8 ± 0.3%, 37.9 ± 3.8% and 64.0 ± 1.7% of intrahepatic hemorrhage for 50, 100 and 200 µg [D-Leu^1^]MC-LR/Kg-treated animals, respectively. In 100 µgMC-LR/Kg-treated animals, the damage was correlated with 25.8 ± 5.6% of intrahepatic hemorrhage, significantly lower than that produced by treatment with [D-Leu^1^]MC-LR at the same dose.

### 2.2. MC-LR and [DLeu^1^]MC-LR Acute Exposure in Phaseolus Vulgaris

The toxic effects of [D-Leu^1^]MC-LR and MC-LR on plants were studied using a single contact exposure model. It consisted of the exposure of *P. vulgaris* seeds at two concentrations (3.5 and 15 µg/mL) of both MCs, only during the imbibition stage (first 24 h).

#### 2.2.1. Effects on Germination, Development and Morphology of Plants

**Figure 3:**
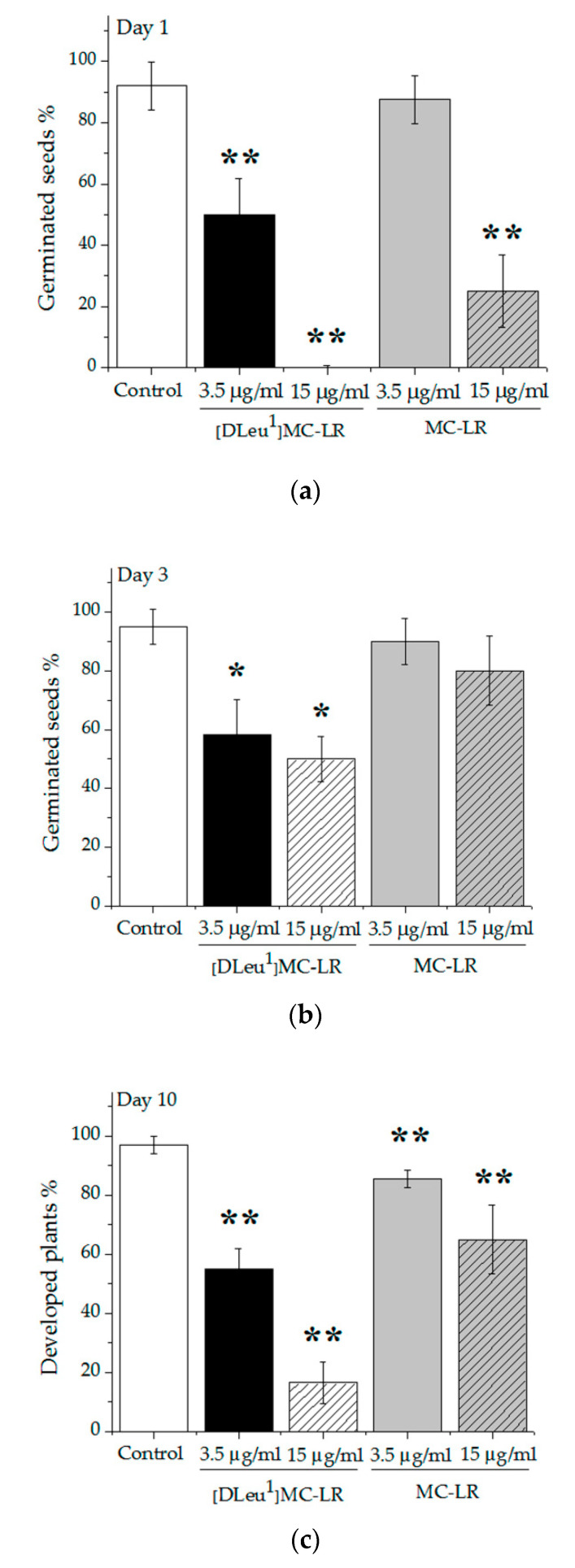
Percentage of *P. vulgaris* germinated seeds (**a**) one day and (**b**) three days after the imbibition stage; and percentage of developed plants (**c**) ten days after the imbibition stage for control group (white bar), 3.5 (black bar) and 15 (dashed bar) µg [D-Leu^1^]MC-LR/mL; and 3.5 (gray bar) and 15 (gray dashed bar) µg MC-LR/mL treatment. The results are expressed as Mean ± SD (*N* = 10) (*) indicates significant differences with respect to control value and (**) indicates significant differences between MC congeners at the same concentration, according to ANOVA test of two independent populations (*p* < 0.05).

Alterations were observed in seed germination and in the number of plants that developed from germinated seeds in treatments carried out with both toxins. One day after the imbibition stage, groups treated with 3.5 or 15 µg [D-Leu^1^]MC-LR/mL and with 15 µg MC-LR/mL showed a decrease in the percentage of seeds able to germinate with respect to control (92.0 ± 7.8%), reaching values of 50.0 ± 11.7% for 3.5 µg [D-Leu^1^]MC-LR/mL and 25.0 ± 11.8% for 15 µg MC-LR/mL (Figure 3a). None of the seeds exposed to 15 µg [D-Leu^1^]MC-LR/mL had been able to germinate at this stage. Three days after the imbibition period, the seeds treated with 3.5 and 15 µg MC-LR/mL showed radicles, reaching 90.0 ± 7.7% and 80.0 ± 11.5% of germinated seeds, respectively (Figure 3b). In the case of seeds treated with 3.5 and 15 µg [D-Leu^1^]MC-LR/mL, although there was an increase in the percentage of germinated seeds with respect to what was observed one day after the imbibition period, the percentage of germinated seeds continued to be significantly lower than the control group after three days (58.0 ± 12.0% for 3.5 µg [D-Leu^1^]MC-LR/mL and 50.0 ± 8.0% for 15 µg [D-Leu^1^]MC-LR/mL).

Ten days after imbibition, the proportion of developed plants from germinated seeds was evaluated. In all groups treated with the studied toxins, some seeds failed to develop a seedling despite having germinated.

The potency of the effect depended on the concentration and the toxin congener used (Figure 3c). Thus, 55.0 ± 7.1%, 16.5 ± 6.9%, 85.4 ± 2.9% and 65 ± 11.8% of plants developed in the case of 3.5 µg [D-Leu^1^]MC-LR/mL, 15 µg [D-Leu^1^]MC-LR/mL, 3.5 µg MC-LR/mL and 15 µg MC-LR/mL, respectively, these percentages being significantly lower than the control group value (97.0 ± 2.8%). Once more we found a greater alteration in the ability to develop seedlings in the groups treated with [D-Leu^1^]MC-LR than in those treated with the same concentration of MC-LR.

**Figure 4:**
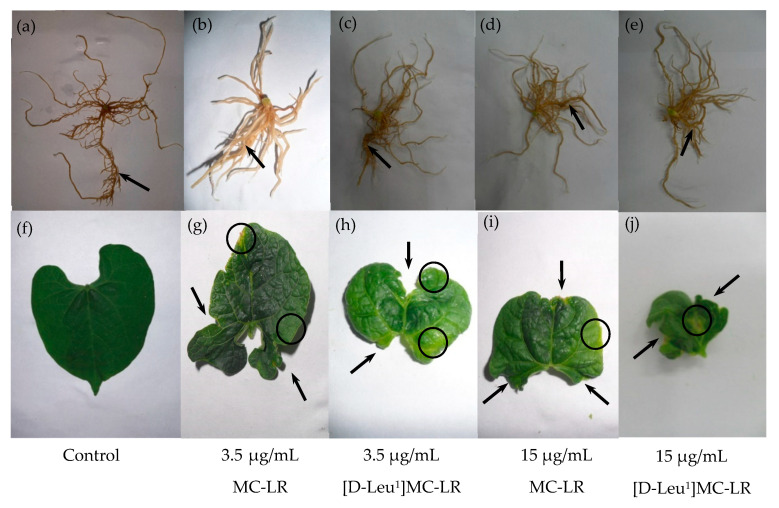
Representative photographs of roots and leaves of control group (**a**,**f**) and groups treated with 3.5 µg MC-LR/mL (**b**,**g**), 3.5 µg [D-Leu^1^]MC-LR/mL (**c**,**h**), 15 µg MC-LR/mL (**d**,**i**) and 15 µg [D-Leu^1^]MC-LR/mL (**e**,**j**). Black arrows indicate the main root or morphological alterations in leaves. Areas with a decrease in pigments are shown by circles.

Morphology alterations in different plant structures developed from seeds treated with [D-Leu^1^]MC-LR or MC-LR were also observed. Figure 4 shows representative photos of roots and leaves of seedlings corresponding to the control group (a, f) and [D-Leu^1^]MC-LR- (c, h, e, j) or MC-LR (b, g, d, i)-treated groups after 10 days of development.

The length of the main root was shorter in the seedlings of all MC treatments (Figure 4b–e) with respect to the control group (Figure 4a). Likewise, a greater development of lateral roots was observed in the roots of these seedlings compared to the control group.

The leaves of the control plants had a homogeneous intense green color and presented a heart shape with smooth edges (Figure 4f). Those treated with 3.5 µg MC-LR/mL (Figure 4g) and 3.5 µg [D-Leu^1^]MC-LR/mL (Figure 4h) were smaller and showed a general alteration in their shape, with rough and irregular edges and chlorosis areas (decrease in pigmentation) on the leaf periphery. These characteristics were intensified in the leaves of plants from groups treated with 15 µg MC-LR/mL (Figure 4i) and 15 µg [D-Leu^1^]MC-LR/mL (Figure 4j).

#### 2.2.2. Biochemical Parameters in Treated Plants

**Figure 5:**
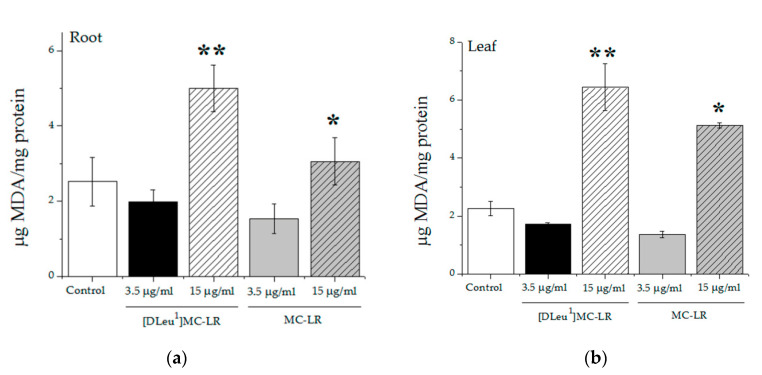
Root (**a**) and Leaf (**b**) lipoperoxide (LOOHs) levels measured as µgMDA/mg protein (TBARs assay) in control group (white bar), 3.5 µg [D-Leu^1^]MC-LR/mL (black bar), 15 µg[D-Leu^1^]MC-LR/mL (dashed bar), 3.5 µg MC-LR/mL (gray bar) and 15 µg MC-LR/mL (gray dashed bar). The results are expressed as Mean ± SD (*N* = 10). (*) represents significantly different from control values and (**) indicates significant differences between MC congeners at the same concentration, according to ANOVA test of two independent populations (*p* < 0.05).

Given that ROS promotion is one of the recognized mechanisms of action of these toxins, we evaluated the levels of lipoperoxides in roots and leaves, the two plant tissues mostly affected by the treatments. The levels of lipoperoxides were significantly higher in leaves (Figure 5b) and roots (Figure 5a) of seedlings from seeds treated with 15 µg MC-LR/mL and 15 µg [D-Leu^1^]MC-LR/mL, while those treated with 3.5 µg/mL of either toxin showed no significant differences with respect to control values. There was a significant difference between the lipoperoxide levels observed in the tissues of plants exposed to 15 µg/mL of both toxins, being higher in plants treated with [D-Leu^1^]MC-LR than in those exposed to the same concentration of MC-LR.

**Figure 6:**
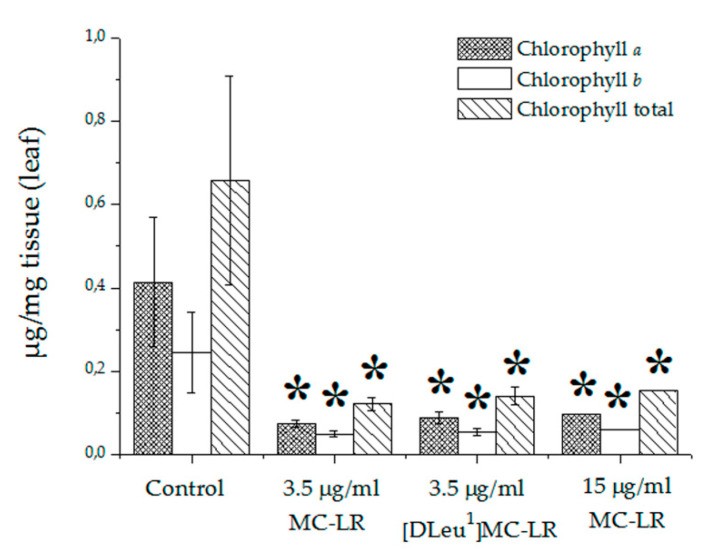
Levels of chlorophyll *a*, *b* and total in leaves of control and [D-Leu^1^]MC-LR- and MC-LR- treated groups. The results are expressed as Mean ± SD (*N* = 10). (*) represents significantly different from control values according to ANOVA test of two independent populations (*p* < 0.05).

Levels of chlorophyll *a*, *b* and total were determined in plant leaves of the control group and groups treated with MCs. Given that for the 15 µg [D-Leu^1^]MC-LR/mL-treated group the percentage of developed plants was the lowest recorded and that the leaves presented the greatest alterations in terms of size and quantity of leaves produced, it was not possible to perform chlorophyll determinations on this group. Significant decreases were observed in chlorophyll *a*, *b* and total levels of plant leaves from 3.5 µg [D-Leu^1^]MC-LR/mL, 3.5 µg MC-LR/mL and 15 µg MC-LR/mL treatments with respect to the control group (Figure 6). However, for this parameter there were no significant differences between the different concentrations of toxin used, or between different toxins at the same concentration.

### 2.3. MCLR and [D-Leu^1^]MC-LR Effects on Protein Phosphatase Activity

We evaluated the inhibitory effects of both toxins on the activity of one of the protein phosphatases identified as a target of their action mechanism (commercial PP1 catalytic subunit) and on the total phosphatase activity present in animal (mice liver) and plant (roots) tissue homogenates.

**Figure 7:**
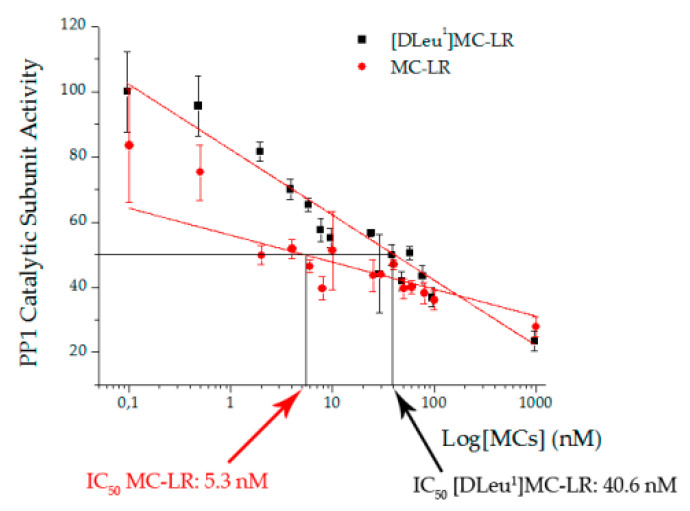
PP1 Catalytic subunit Activity (%) vs. logarithm of [D-Leu^1^]MC-LR (black squares) or MC-LR (red dot) concentration (nM). The results are expressed as Mean ± SD, assessed by triplicate. Note that MC-LR has a lower IC_50_ than [D-Leu^1^]MC-LR.

As expected, PP1 catalytic subunit activity decreased with increasing concentrations of both toxins (Figure 7). However, it was observed that the IC_50_ obtained for MC-LR was lower than that determined for [D-Leu^1^]MC-LR (5.3 nM and 40.6 nM, respectively).

**Figure 8:**
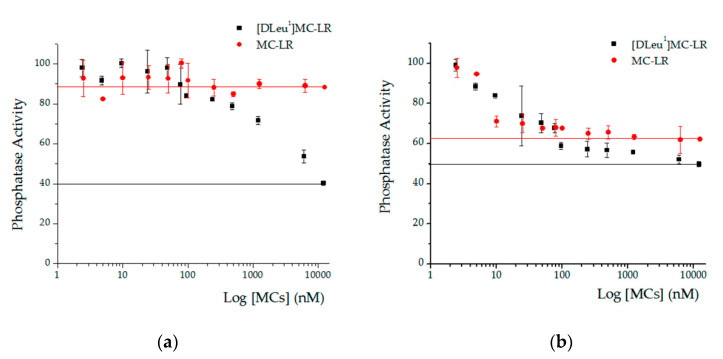
Total phosphatase activity (%) of root (**a**) and liver (**b**) homogenates vs. logarithm of [D-Leu^1^]MC-LR (black squares) or MC-LR (red dots) concentration (nM). Note that in both tissue homogenates, the maximum inhibition induced by [D-Leu^1^]MC-LR was higher than in the case of MC-LR. Horizontal lines indicate the phosphatase activity at the highest [D-Leu^1^]MC-LR (black line) and MC-LR (red line) concentrations employed in the assay. The results are expressed as Mean ± SD, assessed by triplicate.

We observed differences in the maximum level of total phosphatase activity inhibition achieved by [D-Leu^1^]MC-LR and MC-LR in liver and root homogenates (Figure 8). In the case of the evaluation of root homogenate, it was observed that the maximum inhibition produced by [D-Leu^1^]MC-LR (60%) was higher than that generated by MC-LR, which only reached 12% (Figure 8a). The same type of response was observed in the case of liver homogenates, where again, the maximum inhibition of phosphatase activity (50%) was produced by [D-Leu^1^]MC-LR, compared to 40% produced by MC-LR (Figure 8b).

## 3. Discussion

In this paper, we assessed the acute toxic effects of MC-LR and [D-Leu^1^]MC-LR in animal and plant models in order to detect possible differences produced by acute exposure to these toxins. Given the large number of studies addressing the acute toxic effects of MC-LR in animals [4,14,18], we focused on the damage caused by different doses of [D-Leu^1^]MC-LR in mice, for which there is little information available in the literature. We therefore conducted a study on acute exposure in mice to a single administration via i.p. of 25, 50, 100 and 200 µg [D-Leu^1^]MC-LR/kg and 100 µg MC-LR/kg.

Animals treated with 100 µg MC-LR/kg died shortly after the injection, which is in line with previous reports on lethal doses of this toxin [4]. We further observed that all animals treated with 50, 100 and 200 µg [D-Leu^1^]MC-LR/kg also died a few minutes post-injection. On the other hand, none of the animals treated with 25 µg [D-Leu^1^]MC-LR/kg died after the intraperitoneal injection. Although the toxicity test in mice carried out in this study does not constitute a classic LD_50_ determination, the results obtained allow us to infer that [D-Leu^1^]MC-LR LD_50_ could be between 25 and 50 µg/kg, i.e., lower than that reported for MC-LR (50µg/kg) [4]. The histopathological results obtained from 50 to 200 µg [D-Leu^1^]MC-LR/kg of intraperitoneal exposure produced a liver damage pattern characterized by centrilobular intrahepatic hemorrhage, which was also observed in the intraperitoneal administration of 100 µg MC-LR/kg and is in agreement with previously reported descriptions of liver damage derived from acute exposure to MCs [4,13,14,15,18]. Importantly, our findings enabled us to assess the extent of liver damage generated by taking into account the dose and the toxin administered. The data obtained from the different treatments with [D-Leu^1^]MC-LR show that the size of the hemorrhagic area increases with increasing dosage, indicating that the damage in this case is dose-dependent. Similarly, a comparison between the results obtained for the same i.p. dose of MC-LR and [D-Leu^1^]MC-LR (100 µg/kg) showed a larger hemorrhagic area in the liver of animals treated with [D-Leu^1^]MC-LR than in those treated with MC-LR. This indicates that the liver damage generated by these toxins is not only dose-dependent, but also congener-dependent. In addition, the group of mice treated with 25 µg [D-Leu^1^]MC-LR/kg showed a different pattern of damage, characterized by intracytoplasmic microvacuolization that correlates with an initial stage of hepatic steatosis. The appearance of hepatic steatosis has been previously reported in mice treated with an i.p. dose of 25 µg MC-LR/kg but in the context of prolonged exposure with administrations every 48 h for a month (14 total injections) [25]. To the best of our knowledge, there are no reports of a similar alteration produced by the administration of a single 25 µg MC-LR/kg dose.

Information on alterations produced by acute exposure to MCs in plant models is scarce, especially in the case of [D-Leu^1^]MC-LR. The current literature addresses the exposure of different varieties of plants, such as mustard, lettuce, beans, rape, rice, potatoes, etc. by irrigation throughout the study with water with different MC concentrations or with crude extracts containing MCs, especially MC-LR [31,32,33,34,35]. We therefore carried out acute exposure studies in beans (*P. vulgaris*) by single contact during the imbibition stage with relatively high [D-Leu^1^]MC-LR or MC-LR concentrations, in order compare the effects of treatment between the two. As previously discussed, in relation to the toxic effects of acute exposure in an animal model, our findings in a plant model also indicate differences in the toxic potency of [D-Leu^1^]MC-LR with respect to MC-LR. The alterations observed in the germination, development and morphology of the plants differed not only in accordance with the concentration used, but also in terms of [D-Leu^1^]MC-LR vs MC-LR treatment. Seeds treated with [D-Leu^1^]MC-LR showed the greatest delay in germination, requiring three days post-imbibition to emit radicles in the case of those treated with 15 µg/mL, compared to one day for the control group. Furthermore, even though some of the seeds treated with 3.5 µg [D-Leu^1^]MC-LR/mL germinated one day after imbibition and others needed 3 days to achieve germination, 42% of overall treated seeds did not germinate at all. Similarly, 50% of seeds treated with 15 µg [D-Leu^1^]MC-LR/mL failed to germinate. In the case of seeds treated with MC-LR, although a delay in germination of those exposed to 15 µg/mL was observed, on the third day after imbibition the percentage of germinated seeds did not different from the control group and seeds treated with 3.5 µg/mL showed no delay in germination with respect to the control group. The higher toxic potency of [D-Leu^1^]MC-LR was also expressed in terms of greater difficulty in developing germinated seeds. Thus, in the case of [D-Leu^1^]MC-LR treatment, 45% and 83% of germinated seeds failed to develop a seedling 10 days after a single contact with 3.5 and 15 µg/mL of toxin, respectively. In the case of treatment with MC-LR, 14% and 35% of germinated seeds failed to develop seedlings after exposure to 3.5 and 15 µg/mL, respectively. The morphological alterations caused by acute exposure to the two toxins, characterized mainly by rough edges, areas of chlorosis and a reduction in the size of leaves, as well as a decrease in the length of the main root and greater development of secondary roots, are consistent with alterations reported for prolonged exposure treatments by irrigation with different concentrations of MCs [31,32,33,34,35]. In addition, morphological alterations observed in leaves and roots of plants in the present study were found to be dose- and congener-dependent, this being more pronounced in plants grown from seeds treated with [D-Leu^1^]MC-LR than with MC-LR. Alterations observed at the macroscopic level are linked to the findings at the biochemical level, as chlorosis areas observed in leaves could be correlated with decreased chlorophyll levels. Taking into account the increase in lipid peroxidation found especially in the case of higher concentrations (15 µg/mL) of [D-Leu^1^]MC-LR or MC-LR, this decrease could be a consequence of the degradation of chlorophyll in the pro-oxidative environment brought about by treatment with these toxins.

The results of the mouse and bean models indicate that at the same dose or concentration, the toxic effects of [D-Leu^1^]MC-LR are more intense than those of MC-LR. These findings indicate that the different molecular structure, even though it involves the substitution of a single amino acid at position 1 of the molecule, has implications regarding the damage generated by acute exposure to these toxins.

Our results indicate that both toxins generate inhibition of the catalytic subunit of PP1, although the IC_50_ of MC-LR (5.3 nM) is lower than that of [D-Leu^1^]MC-LR (40.6 nM), such that MC-LR produces a more pronounced inhibition of this particular protein phosphatase than that generated by [D-Leu^1^]MC-LR at the same concentration. Though our MC-LR IC_50_ data is consistent with that reported by Mateinsen et al. [5] (MC-LR IC_50_: 3.12 nM; [D-Leu^1^]MC-LR IC_50_: 4.43 nM), we have found differences in terms of [D-Leu^1^]MC-LR characterization. Both our results and those of Matheinsen et al. [5] differ from those reported by Park et al. [7] in terms of the IC_50_ of these toxins (0.3 nM for both toxins). Furthermore, Mateinsen et al. [5] found no differences between the two toxins in terms of the hepatotoxic effects in mice, reporting the minimum lethal dose in both MCs to be 100 µg/kg. Park et al. [7] have proposed the possible implication of these toxins in bird losses in Pakowki Lake, though the presence of these toxins was not monitored as closely as that of other pathogens, such as *Clostridium*, responsible for fowl botulism during bird loss events. In contrast, we have observed that despite the 7-fold higher in vitro PP1 IC_50_ for [D-Leu^1^]MC-LR than for MC-LR, the damage generated by [D-Leu^1^]MC-LR is greater than that caused by MC-LR at the same dose or concentration, both in the animal and plant model of acute exposure. This would indicate that the in vitro PP1 inhibition assay is not a good indicator of MC toxic potency. In line with this, Chen et al. [38] determined in vitro inhibition of PP1 and PP2A generated by eight variants of MCs and subsequently generated a toxicity ranking based on their LD_50_. These results indicate that MC-LR presented the highest toxic potency of the studied MC variants (MC-LR, MC-FR, MC-WR, MC-RR, [D-Asp^3^]MC-FR, [D-Asp^3^]MC-WR, [D-Asp^3^]MC-RR and [Dha^7^]MC-RR), together with the lowest IC50 values for PP1 and PP2A. However, other toxins, such as [D-Asp^3^]MC-FR and [D-Asp^3^]MC-WR, that present a 10- to 12-fold higher IC_50_ for PP1 and 20- to 30-fold higher for PP2A compared to MC-LR IC_50,_ presented a toxic potency similar to that of MC-LR. These three toxins rate highest toxicity based on the LD_50_ ranking of these authors. They also propose that PP2A inhibition may be a better predictor of MC toxicity than PP1 inhibition. However, the rankings established according to the LD_50_ and PP2A IC_50_ coincided in 50% of the toxins studied in the paper. Models [39,40] providing partial explanations for the differences in the chemical bonds established between some varieties of MCs and the site of action of PP1 or PP2A are not applicable to all variants of MCs in the sense of being able to explain the differences in the inhibitory capacity on protein phosphatases [38].

When we analyzed total phosphatase activity inhibition in both liver and root homogenate tissue, we found that [D-Leu^1^]MC-LR had a greater inhibitory effect (liver: 50%, root: 60%) than MC-LR (liver: 40%, root: 12%). In previous studies attributing MC damage to inhibition of PP1 and PP2A phosphatases in different variants of MCs, it was concluded that the particular inhibition of these phosphatases did not determine the toxicity of the toxins [38,41]. Our findings in tissue homogenates provide evidence in this regard, since the significantly more pronounced total phosphatase inhibition generated by [D-Leu^1^]MC-LR is consistent with the greater extent of damage produced by acute exposure to [D-Leu^1^]MC-LR in an animal and plant model. Bearing in mind that a tissue homogenate may be a closer approximation of what occurs when MCs come into contact with a tissue as opposed to an isolated phosphatase, assessment of the effects of MCs on a tissue homogenate could be a more direct reflection of the effects observed in organisms exposed to these toxins. It is likely that the different effects observed in this regard between the two toxins may be due to differences in the ability to inhibit phosphatase activity, not only of the protein phosphatase traditionally associated with the action mechanisms of MCs such as PP1 and PP2A, but also of other enzymes with phosphatase activity present in tissues. In a hyper-phosphorylation scenario, such as that produced by MCs exposure, these differences could be decisive for the fate of the cell affected by the toxins. Along with this, we must take into account that differences in the molecular structure of MCs can affect their mechanisms of absorption, conjugation and elimination, which would also affect the bioavailability of these varieties and, therefore, the extent of the toxic effects produced by each of them.

## 4. Conclusions

Our findings on the evaluation of the toxic effects of acute exposure through a single contact at different doses or concentrations of MC-LR and [D-Leu^1^] MC-LR indicate that, although there is only a slight structural difference between these toxins in terms of the substitution of a single amino acid at position 1 of the molecule, the difference in their toxic potency is relevant. This difference in toxicity was evident both in an animal and a plant model, showing the intensity of damage to be higher for [D-Leu^1^]MC-LR than for MC-LR. The damage generated by these toxins is therefore not only dose- dependent, but also congener-dependent. Biochemical findings such as lipid peroxidation and total phosphatase activity in tissues, corresponding to the main mechanisms of action of these toxins, provide an initial basis to explain the difference in intensity of toxic effects between the two congeners. It is thus necessary to carry out further research into the mechanisms by which the two toxins cause damage. Future studies should be addressed at elucidating which protein phosphatase might be differentially inhibited by [D-Leu^1^]MC-LR; how [D-Leu^1^]MC-LR conjugation and elimination mechanisms are affected; and the effect of exposure conditions to these toxins -in isolation, but also in combination with one another- in animal and plant models, taking into account that they are frequently present simultaneously in blooms present in water bodies of La Plata Basin, constituting a health risk to the population coming into contact with these water bodies.

## 5. Materials and Methods

### 5.1. [D-Leu^1^]MC-LR and MC-LR Purification

**Figure 9:**
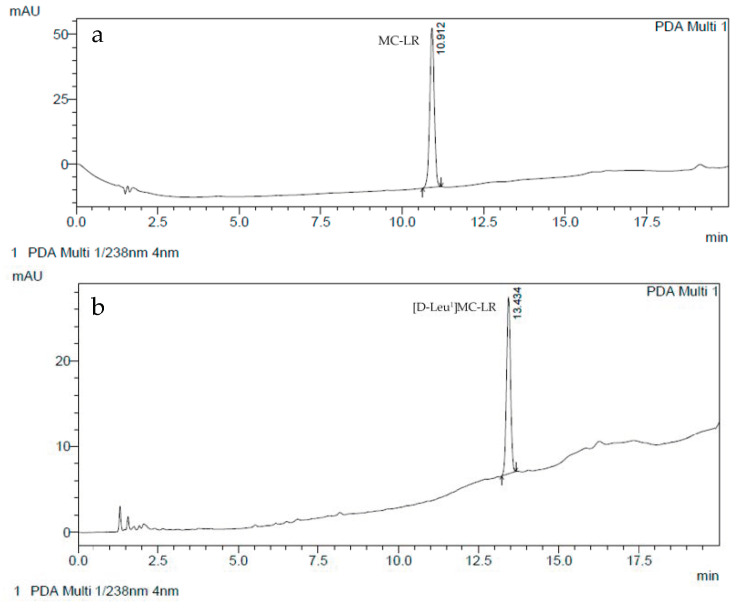
Representative chromatograms of purified MC-LR (**a**) and [D-Leu^1^] MC-LR (**b**) employed in acute exposure assays.

[D-Leu^1^]MC-LR and MC-LR were purified from natural blooms of *Microcystis aeruginosa* collected at the Río de la Plata river, as described in previous works [25,26,28,29]. Briefly, cells were broken by sonication (Omni-Ruptor 400, 15 min) and the extract was treated with chloroform/methanol (50:50; *v*:*v*). The aqueous fraction was concentrated in a rotovap (Decalab, R-23, Buenos Aires, Argentina). Purification was performed with the semi-preparative high-performance liquid chromatography technique. We used a Shimadzu 20A HPLC apparatus with a degassed module and a diode array detector system set at 238 nm. The preparative column used was TERMO Hyperprep HS C18 (250, 10 mm) and the mobile phase was deionized water (TFA 0.05%) with 35% acetonitrile (TFA 0.05%) run in gradient conditions at 5 mL/min. The peak corresponding to [D-Leu^1^]MC-LR or MC-LR was collected separately and concentrated with a previously activated C18 cartridge. Pure [D-Leu^1^]MC-LR and MC-LR were eluted with a solution of methanol:water (90:10, *v*:*v*) after which the methanol was evaporated. Final identification and concentration were achieved by comparison with a Sigma Chemical Inc. (Figure 9) MC-LR standard (St. Louis, MO, USA).

### 5.2. Experimental Design

#### 5.2.1. [D-Leu^1^] MC-LR and MC-LR Acute Exposure in Mice

Twenty-four male N:NIH-S (20–22 g) mice with specific pathogen-free certified status were obtained from the Animal Care Facility Unit of the Veterinary School of Medicine of La Plata National University. They were housed in plastic cages (four animals per cage) and fed ad libitum balanced feed (Alimentos Ganave, Rosario, Argentina) and water. Animals were kept on a 12 h-light/darkness cycle and allowed to acclimatize to their surrounding conditions (well-ventilated room maintained at 23 ± 1 °C) for 1 week before experiments started. Studies were conducted in accordance with international protocols for laboratory animal care and guidelines of local body for protecting animal welfare [42]. The animals experiments were carried out within the framework of the research project entitled: “Cyanobacteria and Cyanotoxins: Scientific and Technological bases for the evaluation of exposure to Microcystins and its removal in water treatment plants”, whose protocols were approved by the National Agency for Scientific and Technological Promotion (ANCyT), on 13 February 2014. The approval code was PICT-2013-0861.

Mice were divided at random into six groups. Four groups were treated with intraperitoneal injection of a single dose of 25, 50, 100 or 200 µg [D-Leu^1^]MC-LR/Kg body weight. Due to the known toxic effect caused by acute exposure by intraperitoneal administration of MC-LR [13,14,15,18] only one group was treated with a single i.p. injection of 100 µg [D-Leu^1^]MC-LR/Kg body weight in order to be able to compare the effect caused by the two toxins. The toxins were prepared freshly by dilution of the stock preparation with saline solution (0.9%; *w*/*v*). The corresponding control group of animals was treated with equivalent volumes of saline solution. Mice behavior was carefully observed after administration of each toxin and the time of death was determined. The mice that did not die, as well as the control group, were euthanized by cervical dislocation 24 h after injection. Liver tissues were dissected and washed with cold PBS. The liver was weighed. Liver tissue from animals treated with [D-Leu^1^]MC-LR or MC-LR and a sub-sample of liver from control animals were fixed for further histological determinations. The rest of the livers from control mice were placed in ice-cold buffered solution (14 mM sodium phosphate, 0.1 mM EDTA, pH 7.40) [43] and homogenized by means of a glass-teflon homogenizer (Kontes Glass Company, Vineland, NJ). Cell debris and nuclei were removed by centrifugation at 10,000× *g* (20 min at 1–2 °C) in a Sorvall RC5C Dupont centrifuge (Newtown, CT). Supernatants were aliquoted and frozen in hermetic polypropylene vials at −70 °C under N_2_ atmosphere until used for total phosphatase activity measurements.

#### 5.2.2. [D-Leu^1^] MC-LR and MC-LR Acute Exposure in Beans

Five groups of ten healthy-looking *Phaseolus vulgaris* seeds were employed for MC acute exposure evaluation. The toxins were prepared freshly by dilution of the stock preparation with sterile water free of MCs. Two groups of seeds were exposed to 3.5 and 15 µg/mL of [D-Leu^1^]MC-LR and another two groups were exposed to 3.5 and 15 µg/mL of MC-LR. A corresponding control group of seeds was treated with equivalent volumes of sterile water free of MCs. Acute exposure to toxins was achieved by contact of the seeds with [D-Leu^1^]MC-LR or MC-LR only during the imbibition stage. Each seed was placed in a test tube with 1 mL of the appropriate MC-LR, [D-Leu^1^]MC-LR solution or sterile MCs-free water. The tubes were placed in a temperature chamber (24 ± 1 °C) for 24 h (imbibition stage). Then, each seed was sown in sterile sand moistened with sterile MCs-free water and maintained in a temperature chamber (24 ± 1 °C), 14 h-light/dark cycles and 42 μmol photons/m^2^s light intensity for 10 days to allow for germination and subsequent seedling development. The seeds were irrigated daily with sterile MC-free water and macroscopic observation was carried out throughout the test. Ten days after the imbibition stage, leaves and root samples of the developed plants were taken. The roots were washed with sterile water and the leaves and roots photographed. One leaf of each plant of the different treatments was processed for chlorophyll determination. Roots and leaves were placed in an ice-cold buffered solution (20 mM sodium phosphate, pH 7.0) and homogenized by means of a mortar. Cell debris and nuclei were removed by centrifugation at 10,000× *g* (20 min at 1–2 °C) in a Sorvall RC5C Dupont centrifuge (Newtown, CT). Supernatants were aliquoted and frozen in hermetic polypropylene vials at −70 °C under N_2_ atmosphere until used for analytical measurements.

### 5.3. Analytical Determinations

#### 5.3.1. Histological Studies

Liver sections were fixed in 25 volumes of 10% formalin in phosphate-buffered saline (PBS), pH 7. Tissue samples were dehydrated in an ethanol gradient, placed in chloroform to replace the ethanol and then placed in liquid wax. Sections (4–5 mm thick) were stained with hematoxylin and eosin (H&E) [44]. The slices were analyzed under optical microscope (Olympus Binocular Microscope) and imaged for the morphometric analysis. ImageJ software (free access software) was used to carry out the morphometric analysis. In order to quantify hepatic hemorrhage, we determined the area occupied by blood infiltration (hemorrhagic area) and the total area of tissue in each photograph. The ratio hemorrhagic area/total area was expressed as a percentage.

#### 5.3.2. Biochemical Parameters Determined in Roots and Leaves

To determine lipid peroxidation, samples from roots and leaves homogenates were processed for thiobarbituric acid reactive substances (TBARs) and measured as malondialdehyde (MDA). The colorimetric method described by Okawa et al. [45] was used.

Leaf chlorophyll levels were determined according to the method described by Lichtenthaler et al. [46]. Briefly, the leaves were placed in methanol and kept at −20 °C and dark overnight. The cell debris was then separated by centrifugation 5000 rpm in Rolco centrifuge and the colorimetric determination was made in the supernatant according to the cited method, using the following equations:

Chl*_a_* = 16.72 × A_665_ − 9.16 × A_652_ (1)

Chl*_b_* = 34.09 × A_652_ − 15.28 × A_665_ (2)

Chl *_total_* = 1.44 × A_665_ − 24.93 × A_652_ (3)

#### 5.3.3. Protein Phosphatase Activity Assay

PP1 catalytic subunit was purchased form Sigma Chemical Inc. The protein phosphatase activity assay was carried out on PP1 catalytic subunit and on control liver and root homogenates following the protocol described by Chen et al. [38]. Briefly, the commercial PP1 enzyme was diluted according to the manufacturer’s instructions with 50 mM Tris–HCl buffer containing 0.1 mM EDTA, 5 mM dithiothreitol, 0.2 mM MnCl_2_ and 0.2 mg/mL bovine serum albumin at pH 7.0. Livers of mice and roots of *P. vulgaris* seedlings having no contact with MCs were homogenized in the same ice-cold buffer. Para-nitrophenyl phosphate (p-NPP), diluted in the buffer described above, was employed as substrate. One unit (U) was defined as the phosphatase activity that hydrolyzes 1 nmol of para-Nitrophenyl phosphate (p-NPP) in 1 min. The phosphatase assay was carried out in 96-well microplates. In each case 100 µL Tris–HCl buffer was added and mixed with aqueous solution with different amounts of [D-Leu^1^]MC-LR or MC-LR and 50 µL of commercial enzyme solution or tissue homogenate. The reaction was pre-incubated for 15 min. at 30 °C, after which 50 µL of substrate solution was added and the reaction kept at 30 °C during one hour. For each experiment, p-NPP blank reaction (without toxins and enzyme or homogenates) and homogenates blank reaction (without toxins and p-NPP) were also carried out. After stopping the reaction with 0.5 N NaOH solution, absorbance was determined at 405 nm. The phosphatase activity in each case was calculated by the change in absorbance and expressed as a percentage activity of the control.
